# A Simple Overview of Pancreatic Cancer Treatment for Clinical Oncologists

**DOI:** 10.3390/curroncol30110694

**Published:** 2023-10-31

**Authors:** Ingrid Garajová, Marianna Peroni, Fabio Gelsomino, Francesco Leonardi

**Affiliations:** 1 Medical Oncology Unit, University Hospital of Parma, 43125 Parma, Italy; marianna.peroni@unipr.it (M.P.);; 2 Department of Oncology and Hematology, University Hospital of Modena, 41124 Modena, Italy

**Keywords:** pancreatic cancer, therapy, prognosis

## Abstract

Pancreatic cancer (PDAC) is one of the most aggressive solid tumors and is showing increasing incidence. The aim of our review is to provide practical help for all clinical oncologists and to summarize the current management of PDAC using a simple “ABC method” (A—anatomical resectability, B—biological resectability and C—clinical conditions). For anatomically resectable PDAC without any high-risk factors (biological or conditional), the actual standard of care is represented by surgery followed by adjuvant chemotherapy. The remaining PDAC patients should all be treated with initial systemic therapy, though the intent for each is different: for borderline resectable patients, the intent is neoadjuvant; for locally advanced patients, the intent is conversion; and for metastatic PDAC patients, the intent remains just palliative. The actual standard of care in first-line therapy is represented by two regimens: FOLFIRINOX and gemcitabine/nab-paclitaxel. Recently, NALIRIFOX showed positive results over gemcitabine/nab-paclitaxel. There are limited data for maintenance therapy after first-line treatment, though 5-FU or FOLFIRI after initial FOLFIRINOX, and gemcitabine, after initial gemcitabine/nab-paclitaxel, might be considered. We also dedicate space to special rare conditions, such as PDAC with germline BRCA mutations, pancreatic acinar cell carcinoma and adenosquamous carcinoma of the pancreas, with few clinically relevant remarks.

## 1. Introduction

Historically, pancreatic cancer has been associated with aggressive behavior, poor prognosis and low survival rates that have remained relatively unchanged over the past decades (5-year overall survival rate of approximately 5–10%) [1,2]. It is among the ten most cruel solid tumors, and nowadays, it represents the seventh leading cause of cancer-related deaths worldwide. Its incidence is estimated to rise in the following years [3,4,5,6]. Its poor prognosis is mainly due to early systemic spread, local aggressiveness and the poor efficacy of actual treatments. PDAC arises through multiple steps from precursor lesions to undergoing progression from low-grade to high-grade dysplastic lesions and acquiring increasing cytological atypia and genetic aberrations [7,8,9]. Generally, two main types of PDAC precursor lesions are recognized: pancreatic intraepithelial neoplasias (PanINs, 85–90%) and cystic lesions of the pancreas. Cystic pancreatic lesions include intraductal papillary mucinous neoplasms (IPMNs) and mucinous cystic neoplasms (MCNs) [7,8,9]. Generally, cystic neoplasms of the pancreas can be diagnosed via imaging techniques; on the contrary, the detection of PanINs is not possible using abdominal imaging scans [10]. Up to 10% of pancreatic cancer patients have a germline predisposition to malignancy. The vast majority of these patients harbor somatic mutations in four commonly altered genes, namely, KRAS in approximately 90% of PDACs, TP53 in 80%, CDKN2A in 60% and SMAD4 in 40%, followed by a series of genes altered significantly more often than expected by chance [9]. The majority of PDAC patients are diagnosed with advanced disease, and only a small percentage of patients are eligible for surgical resection. Early stage diagnosis is difficult since PDAC patients suffer non-specific symptoms, except for tumors of the pancreatic head, which might lead to jaundice even in the early stage, as a consequence of the obstruction of the common bile duct. Other symptoms include epigastric pain with posterior irradiation, impaired general condition, steatorrhea and new-onset diabetes. The most common pancreatic cancer is ductal adenocarcinoma (PDAC), which accounts for more than 90% of all pancreatic malignancies. Radiological staging should initially include computed tomography (CT) with arterial and portal phases in order to assess local vessel involvement and to determine the precise tumor size and burden; the general appearance of pancreatic cancer on CT is a hypoattenuating homogeneous mass with indistinct margins in the arterial phase. Magnetic resonance imaging (MRI) is particularly useful for the detection of hepatic lesions that cannot be precisely characterized via CT. Endoscopic ultrasound (EUS) today represents the gold standard for pancreatic cancer initial workups. EUS enables doctors to obtain the pancreatic cancer tissue necessary for histological characterization via fine-needle aspiration (FNA), though this technique also permits biliary stenting if required, in particular, with fully covered self-expanding metal stents [11,12]. Some experts suggest the use of explorative laparoscopy to exclude peritoneal carcinosis, in cases of unclear or suspicious radiological imaging in non-metastatic pancreatic tumors, in order to offer surgical evaluation to all non-metastatic PDAC patients. However, this approach is not generally recommended, especially in patients with high-risk features. Similarly, positron emission tomography (PET) is not actually recommended as a part of the initial staging workup as, for the majority of PDAC patients, it does not add more information compared to CT or MRI imaging [13]. Before starting any systemic treatment, it is mandatory to confirm satisfactory blood parameters, such as complete blood counts, hepatic and renal function and the baseline value of tumor blood biomarkers, such as carbohydrate antigen 19-9 (CA 19-9), which represents the most useful tumor marker in pancreatic cancer and provides prognostic information. For patients with resectable disease, elevated preoperative CA 19-9 levels predict poorer survival post-resection and a failure to normalize, and the elevation of postoperative CA 19-9 levels has been recognized to predict the recurrence of PDAC [14]. Moreover, CA 19-9 elevations have been shown to precede clinical or radiological recurrence by 2–6 months [15]. When chemotherapy with 5-fluorouracile (5-FU) or capecitabine is planned, a dipyrimidine dehydrogenase (DPD) deficiency test and cardiologic evaluation should be performed [16]. Patients’ general condition, past medical history and nutritional status are all important parameters to take into account when defining the type of treatment. After the completion of the initial staging workup, pancreatic cancer is divided, according to its resectability, into resectable, borderline resectable, locally advanced or metastatic. TNM classification is less often used. It is important to underline that each patient affected by pancreatic cancer has to be discussed by a multidisciplinary board (including oncologists, hepatobiliary or pancreatic surgeons, radiologists, endoscopists, pathologists and radiotherapists) before making a definitive therapeutic plan, especially for localized tumors without distant metastases.

## 2. Resectable PDAC and Simple “ABC Method”

**Figure 1:**
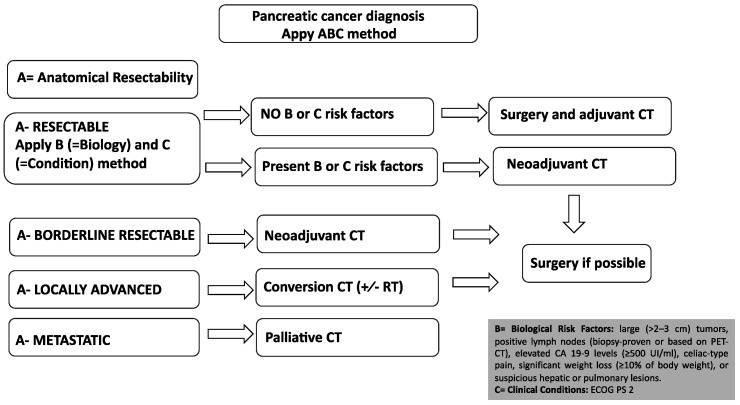
Treatment algorithm for PDAC patients using “ABC method” (A—anatomical resectabi- lity, B—biological resectability and C—clinical conditions). All PDAC patients should initiate systemic treatment apart from anatomically resectable PDAC without high-risk biological factors (absence of B factors) and fitness for surgery (absence of C factor), as demonstrated in the figure. In all cases, there are no rigid cut-offs for any high-risk aggressive features; therefore, these are left as considerations for the treating oncologists/surgeons. Note that before any systemic chemotherapy, biopsy for histological characterization is mandatory. High-risk biological features include suspicious hepatic or pulmonary lesions, the presence of positive lymph nodes (histologically proven or based on positivity of PET-FDG scan), large pancreatic primary tumors with dimension superior to 2–3 cm, elevated baseline level tumor biomarker CA 19-9 and several clinical characteristics such as celiac-type pain or significant weight loss (≥10% of body weight).

Surgical resection is the only potentially curative treatment for pancreatic cancer. Unfortunately, only a minority of PDAC patients (approximately 15–20%) are judged to be technically eligible for potentially radical resection at the time of initial diagnosis. Anatomical resectability can be determined according to different international guidelines. The most used international classifications are MD Anderson Cancer Center, AHPBA/SSO/SSAT, NCCN and Alliance [17,18,19,20]. Generally, resectable PDAC is defined as the absence of arterial and venous contact. Regarding arterial involvement, an exact description of the common hepatic arteries, celiac axis or superior mesenteric artery is necessary, and for venous involvement, the portal and superior mesenteric vein are of particular interest to pancreatic surgeons. It needs to be specified that NCCN and Alliance guidelines consider venous contact (without arterial involvement) <180° as anatomically resectable PDAC if there is no deformity or stenosis of the portal and superior mesenteric vein [17,18,19,20]. The major aim of surgery is its radicality in terms of R0 margin achievement. R0 margins are characterized by a distance >1 mm between tumor cells and margins. The type of surgery depends on the primary tumor location: the recommended surgery in the case of pancreatic head tumors is pancreatoduodenectomy, also known as the Whipple procedure. In the case of cancer of the body and tail, distal pancreatectomy with splenectomy should be performed. As for the other gastrointestinal solid tumors, a minimum number of lymph nodes should be removed during surgery; for PDAC, a minimum of 15 lymph nodes is recommended. Another important point in PDAC diagnostics and treatment workup is biopsy. If upfront resection is planned, no histological characterization is necessary in the case of radically resectable pancreatic tumors without high-risk features, with typical radiological and clinical imaging. In the presence of jaundice, preoperative biliary stenting is not necessary in order to normalize blood levels of bilirubin, and upfront surgical resection should be recommended. This is to avoid complications associated with biliary drainage that may delay the surgery. Only when jaundice is associated with cholangitis or bilirubin > 25 mol/L is preoperative biliary stenting considered [21]. High-volume centers for pancreatic surgery are recommended as these procedures are burdened by the high rates of mortality and morbidity. It is very important to pay attention to possible postoperative complications, such as steatorrhea, where, for pancreatic enzymes, a prescription might be indicated (see also the next chapter on pancreatic exocrine insufficiency). Moreover, glucose blood levels should be monitored and adequate treatment for diabetes should be prescribed. Finally, splenectomy renders the patients more vulnerable to infectious complications; therefore, vaccinations, such as meningococcus and pneumococcus, should be performed. Unfortunately, even if radical surgery is possible with R0 margins, the rate of recurrence for pancreatic cancer patients is very high, particularly during the first 2 years. The prevalent way of disease recurrence is systemic with distant metastases, suggesting that patients might hide micro-metastatic disease [2,22]. In order to reduce the risk of high recurrence, adjuvant chemotherapy should be offered to all PDAC patients who undergo pancreatic surgery for cancer. For fit patients, an adjuvant three-drug chemotherapy regimen with modified FOLFIRINOX (fluorouracil, irinotecan, leucovorin, and oxaliplatin) represents the actual standard of care in this setting. This regimen showed a significant improvement in median overall survival (OS) compared to gemcitabine monotherapy (OS 54.4 vs. 35 months, HR 0.64; *p* = 0.003) [23]. Gemcitabine plus capecitabine or gemcitabine alone are alternatives for patients with contraindications to FOLFIRINOX, such as patients over 75 years old, with poor performance status or postoperative complications [24,25,26,27]. The median OS for PDAC patients treated after potentially radical pancreatic surgery with gemcitabine plus capecitabine was 28.0 months compared with 25.5 months in the gemcitabine group (HR 0.82; *p* = 0.032) [25]. Adjuvant chemotherapy with gemcitabine in monotherapy showed better efficacy in terms of longer DFS (13.4 vs. 6.9 months in the control arm; *p* < 0.001), though no statistically significant difference in OS was seen (22.1 vs. 20.2 months in the control arm; *p* = 0.06) [26]. Interestingly, in the subgroup analysis, only PDAC patients with negative lymph nodes on the final histological report had statistically significant benefits in terms of OS from adjuvant gemcitabine treatment (34 vs. 27.6 months; *p* = 0.04) [26]. Adjuvant chemotherapy should generally start within 12 weeks from surgical resection, even though evidence suggests that the completion of adjuvant treatment, rather than its timing, is important to achieve an OS benefit [28,29]. On the contrary, adjuvant chemoradiotherapy is not currently indicated and should not be performed outside of clinical trials [30]. The clinical evidence for neoadjuvant treatment use for anatomically resectable PDAC is mostly based on the long-term results of the PREOPANC trial and a meta-analysis of randomized controlled clinical trials, in which both resectable and borderline resectable PDAC patients were evaluated [31,32]. Therefore, neoadjuvant approaches in clearly resectable PDAC without high-risk factors are still contradictory, and the results of phase III trials are awaited (PREOPANC3 and ALLIANCE A021806) [33,34]. Through the years, the definition of resectable PDAC has been updated to achieve a more uniform definition, and in addition to “anatomic considerations”, “biological” and “conditional”, factors are now being considered to define pancreatic cancer to be resectable or borderline resectable (“ABC method”) [35]. Therefore, even patients with anatomically resectable PDAC should be evaluated for the presence of high-risk biological and conditional features. High-risk biological features include suspicious hepatic or pulmonary lesions, the presence of positive lymph nodes (histologically proven or based on the positivity of a PET-FDG scan), large pancreatic primary tumors with dimensions superior to 2–3 cm, elevated baseline levels of the tumor biomarker CA 19-9 and several clinical characteristics, such as celiac-type pain or significant weight loss (≥10% of body weight) [16,35]. In fact, increased baseline serum levels of CA 19-9 (especially ≥500 UI/mL) inversely correlate with resectability as well as with survival rates [36]. Similarly, the existence of positive lymph nodes strongly impacts the prognosis of PDAC patients regardless of tumor resectability or tumor stage [37]. The high-risk conditional factors include performance status and co-morbidities that increase the risk of morbidity or mortality after surgery. Given the higher probability of non-radical resection, patients with resectable tumors and high-risk factors are not considered optimal candidates for upfront surgery and should, therefore, receive neoadjuvant chemotherapy (Figure 1). Neoadjuvant chemotherapy includes several advantages, such as the early treatment of micrometastatic disease, the downstaging of disease and an increased likelihood of an R0 resection, and is associated with increased OS rates; in addition, it does not negatively affect major surgical complications rates. Before neoadjuvant treatment initiation, a biopsy for PDAC confirmation is mandatory. According to the SWOG S1505 trial, which compared a neoadjuvant chemotherapy three-drug regimen with modified FOLFIRINOX and a neoadjuvant two-drug regimen with gemcitabine/nab-paclitaxel in patients with anatomically resectable pancreatic cancer, no statistically significant difference in overall survival was seen between the two treatment arms, though the gemcitabine/nab-paclitaxel regimen showed a higher rate of complete and major pathologic response (42% vs. 25% in the mFOLFIRINOX arm) [38]. The optimal duration of neoadjuvant treatment is unknown. There is a general consensus that 6 months of nonsurgical therapy is optimal; therefore, adjuvant chemotherapy can be administered continuing the preoperative regimen for a total of 6 months.

## 3. Borderline Resectable and Locally Advanced PDAC

Borderline resectable pancreatic adenocarcinomas (BRPCs) are a subgroup of technically resectable pancreatic cancer but at high risk of non-radical resection (R1) and/or early recurrence after surgery (anatomically resectable PDAC with high-risk biological or conditional factors). Locally advanced pancreatic adenocarcinomas (LAPCs) are tumors with local infiltration that preclude potentially radical pancreatic surgery with R0 margins. Both BRPC and LAPC should, therefore, be treated with initial systemic chemotherapy treatment using the same regimens used in a metastatic setting (FOLFIRINOX or gemcitabine/nab-paclitaxel; see Figure 1) [39,40]. According to a recent meta-analysis of non-randomized patient cohorts, in patients with BRPC or LAPC, primary treatment with FOLFIRINOX compared with gemcitabine-based chemotherapy appears to provide a survival benefit for patients that are ultimately unresectable [41]. For patients who undergo surgical resection, outcomes are similar between gemcitabine-based chemotherapy and FOLFIRINOX when delivered in a neoadjuvant setting [41]. When the tumor is not resectable after induction chemotherapy, chemoradiotherapy might be considered, even if several randomized studies did not demonstrate any survival benefit and its role remains controversial [42,43]. Less than 30% of LAPC will undergo surgery after “conversion therapy” [43,44], and one-third of PDAC patients die from local progression without distant metastases. Finally, chemoradiotherapy can be useful as a symptomatic treatment of LAPC-related pain [16,45].

## 4. Metastatic PDAC

### 4.1. First-Line Therapy

According to phase III clinical trials, FOLFIRINOX or gemcitabine/nab-paclitaxel are the two regimens for fit PDAC metastatic patients [39,40]. FOLFIRINOX is a three-drug regimen that showed better survival outcomes compared to gemcitabine monotherapy with an OS of 11.1 vs. 6.8 months, respectively, and is recommended for metastatic PDAC patients with good clinical conditions (ECOG PS 0–1) and younger ages (less than 75 years). The median PFS was 6.4 months in the FOLFIRINOX group and 3.3 months in the gemcitabine group. Progressive disease was described in 15.2% in the FOLFIRINOX group and 34.5% in the gemcitabine group [39]. Importantly, according to a meta-analysis that evaluated 1461 metastatic PDAC patients, no difference in mOS, mPFS, and ORR between the standard FOLFIRINOX regimen utilized in the PRODIGE4 study and the modified FOLFIRINOX regimens was seen. Modified FOLFIRINOX differs from “classical” FOLFIRINOX in the omission of the 5-FU bolus and/or dose reductions in infusional 5-FU, irinotecan, and/or oxaliplatin, with consequent less collateral side effects and better tolerance. Based on these studies, modified FOLFIRINOX regimens have been adopted in clinical practice for first-line palliative settings [46]. Gemcitabine and nab-paclitaxel represent an alternative regimen for metastatic PDAC patients, as OS in the gemcitabine–abraxane group improved with statistical significance compared to gemcitabine monotherapy (8.5 vs. 6.7 months, *p* < 0.001). The median PFS was 5.5 months in the gemcitabine and nab-paclitaxel group and 3.7 months in the gemcitabine group. Progressive disease was described in 20% in the gemcitabine and nab-paclitaxel group and 26% in the gemcitabine group [40,47]. There are no prospective randomized trials with a head-to-head comparison between the two regimens (FOLFIRINOX vs. gemcitabine/nab-paclitaxel), though analyses of non-randomized “real world” studies to date have not provided evidence of a major benefit of one regimen over the other; therefore, there is no clear preference [48]. Recent studies have suggested that basal-like PDAC characterized by low GATA-6 tissue expression is less sensitive to platinum-based chemotherapy (including FOLFIRINOX) than the classical type [49,50]. Single-agent gemcitabine can be given to patients with poorer performance status to provide clinical benefit [51]. Clinical benefit response is experienced by 23.8% of gemcitabine-treated patients compared with 4.8% of 5-FU-treated patients. The median survival durations were 5.6 and 4.4 months for gemcitabine-treated and 5-FU-treated patients, respectively. The survival rates at 12 months were 18% for gemcitabine patients and 2% for 5-FU patients [51]. A PS of 3 or 4, however, contraindicates any palliative chemotherapy and only allows the best supportive care. Therefore, PDAC patients who are capable of only limited selfcare, confined to a bed or chair more than 50% of waking hours, should not undergo any active anticancer treatment. Recently, at the ASCO 2023 conference, the results of phase III NAPOLI-3 were shown. In this study, metastatic PDAC patients were randomized to receive first-line NALIRIFOX versus gemcitabine and nab-paclitaxel. In the NALIFIROX-treated participants, the median OS was 11.1 vs. 9.2 months in the gemcitabine plus nab-paclitaxel group, resulting in statistical significance. A significant improvement was also observed in PFS (7.4 months for NALIFIROX vs. 5.6 months for gemcitabine plus nab-paclitaxel). Progressive disease was observed for 9.9% in the NALIRIFOX group vs. 14.5% in the gemcitabine and abraxane group [52].

### 4.2. The Role of Maintenance after First-Line Therapy

There are limited data to recommend the management of patients with locally advanced or metastatic pancreatic cancer who achieved disease control or reduction after first-line palliative treatment. In this setting, maintenance therapy represents an important tool in order to minimize chemotherapy toxicity while preserving survival benefits [53]. The only drug approved for maintenance therapy in metastatic PDAC, with germline BRCA mutation, is Olaparib [54], though it is not available in all countries due to regulatory restrictions. Generally, after FOLFIRINOX, maintenance with 5-FU/capecitabine or FOLFIRI might be considered. 5-FU monotherapy maintenance appeared to be as effective as FOLFIRI, in a FOLFIRINOX de-escalation maintenance strategy; therefore, 5-FU maintenance might be considered an option after 4 months of FOLFIRINOX chemotherapy with no progressive disease [55,56]. After gemcitabine and nab-paclitaxel, maintenance with gemcitabine might be considered [57]. The optimal maintenance strategy, however, is not defined.

## 5. Second-Line Therapy

**Figure 2:**
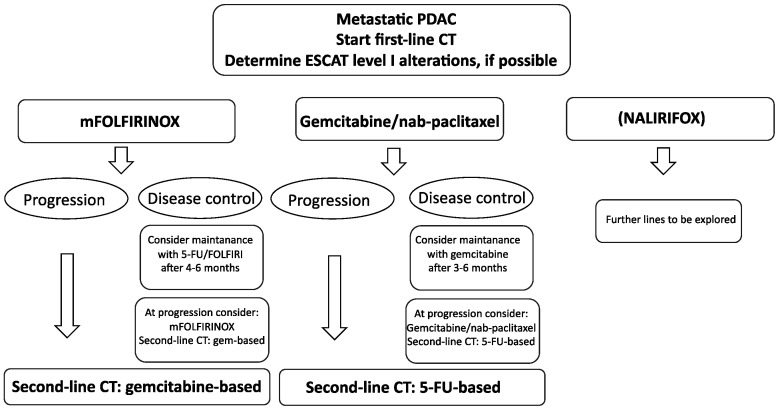
Treatment algorithm for metastatic PDAC. FOLFIRINOX and gemcitabine/nab-paclitaxel are the current standard of care in first-line settings. If patients are initiated with FOLFIRINOX with disease control, consider maintenance therapy with 5-FU/FOLFIRI after a minimum of 4 months of FOLFIRINOX. If patients are initiated with gemcitabine/nab-paclitaxel with disease control, consider maintenance therapy with gemcitabine after a minimum of 3 months of gemcitabine/nab-paclitaxel. Second-line chemotherapy changes the backbone: initial treatment with FOLFIRINOX is gemcitabine-based, while initial treatment with gemcitabine/nab-paclitaxel is 5-FU-based. Where possible, determine the three ESCAT level I alterations (germline BRCA1/2, MSI-high, NTRAK).

**Figure 3:**
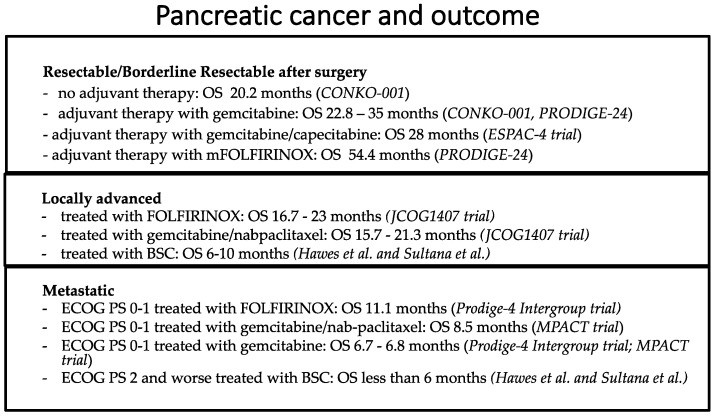
Outcome of patients affected by resectable/borderline resectable, locally advanced and metastatic PDAC. For metastatic PDAC patients, the OS outcome is less than 1 year; for locally advanced PDAC patients, the OS outcome is less than 2 years; for resectable PDAC patients who have undergone adjuvant FOLFIRINOX, an OS of 54 months has been reported [23,25,26,40,41,71].

Around half of metastatic PDAC patients are eligible for second-line chemotherapy [58]. Currently, there is no standard second-line treatment. Generally, after progression on FOLFIRINOX, treatment with gemcitabine, as well as gemcitabine and nab-paclitaxel, is a reasonable option, when feasible considering the regulatory issues in different countries [59,60,61]. After progression on first-line gemcitabine-based chemotherapy regimens, several treatment possibilities with 5-FU-based combinations are available, including liposomal irinotecan plus 5-FU/leucovorin combinations, FOLFIRI, mFOLFIRINOX, FOLFOX, XELOX, OFF (oxaliplatin, 5-FU/LV) or docetaxel plus oxaliplatin combinations [62,63,64,65,66,67,68,69]. According to a recent meta-analysis, irinotecan-based regimes (NALIRI and FOLFIRI) may be the preferred options for second-line treatment with regard to survival outcomes, as shown in Figure 2 and Figure 3 [70].

### 5.1. Management of Pancreatic Exocrine Insufficiency and Other Potential Side Effects and Complications of Surgery and Systemic Treatments

Pancreatic exocrine insufficiency and other potential complications of surgery.

The prevalence and severity of pancreatic exocrine insufficiency (PEI) depend mainly on the location of the primary pancreatic tumor, the disease stage and the type of surgery. The expected PEI prevalence is high if the primary tumor is localized in the pancreatic head in both resected and unresectable PDACs (70% vs. 85%, retrospectively) when compared to primary body/tail pancreatic cancer localization (around 30% for both resectable and unresectable PDACs) [72]. Pancreatic enzyme replacement therapy (PERT) is the mainstay of PEI management to reduce nutritional deficiencies. Pezzilli et al. [72] suggested a pragmatic approach for testing and treating PEI in PDAC patients: (a) start PERT in all PDAC patients if the primary tumor is localized in the pancreatic head and there are symptoms or signs of malabsorption, independent of disease stage; (b) test for PEI by means of fecal-elastase-1 (FE-1) levels in patients with a tumor in the body or tail, and start PERT only in cases of symptoms of maldigestion or in the presence of low values of FE-1 (<200 μg/g). The clinical suspicion of PEI includes steatorrhea, flatulence, bloating, urgency and abdominal discomfort or post-prandial abdominal pain. The recommended initial dose of pancreatic extract is 40,000–50,000 lipase units per meal (4 cp of Creon 10.000 UI during lunch and dinner) and 25,000 lipase units per snack (2 cp of Creon 10.000 UI during breakfast), and this dose should be increased until the steatorrhea is sufficiently reduced. Importantly, Creon capsules should be taken during meals or snacks (not before or after), with sufficient fluid intake. Creon capsule contents should not be crushed or chewed but should be swallowed whole. This dosage should be maintained over time. Further, for resected PDAC patients, glycemia should be monitored, and for patients who have undergone splenectomy, vaccinations (meningococcus and pneumococcus) are recommended [16]. Postoperative complication rates range between 24.3% and 64%, and most frequently, patients suffer from pancreatic fistula, abdominal abscess, hemorrhage, bile or enteral anastomosis leakage [73].

### 5.2. Potential Side Effects of Systemic Treatments

Regarding potential treatment toxicities, patients treated with the nab-paclitaxel–gemcitabine suffer, most frequently, nonhematologic adverse events, such as fatigue (in 54% of patients), alopecia (in 50% of patients) and nausea (in 49% of patients). Treatment-related adverse events of grade 3 or higher are neutropenia (38%), fatigue (17%) and peripheral neuropathy (17%). The incidence of febrile neutropenia is 3%. Fatal events are reported for 4% of patients [40]. Patients treated with FOLFIRINOX with palliative intent have a higher incidence of grades 3 or 4 neutropenia (45.7%), febrile neutropenia (5.4%), thrombocytopenia (9.1%), diarrhea (12.7%) and sensory neuropathy (9%), as well as grade 2 alopecia (11.4%) [39]. Further, an important issue in PDAC is thromboprophylaxis. There has been reported a high prevalence rate of venous thromboembolism in PDAC, which, therefore, has to be considered. The occurrence of thrombotic events is about 25% in PDAC patients. The mechanism is multifactorial, but systemic chemotherapy treatment increases the risk. Three trials have demonstrated the safety and efficacy of primary prevention for venous thromboembolic events with prophylactic LMWH, apixaban or rivaroxaban in outpatients with advanced PDAC undergoing chemotherapy [74,75,76].

## 6. Oligometastatic Disease

The evidence on the surgical management of oligometastatic PDAC is scarce. However, some evidence exists that resection of metachronous liver and pulmonary metastases can be performed safely and should be considered as it may be superior to palliative treatment [77,78,79]. The survival benefit is less clear in synchronous metastases, though initiation with systemic treatment and the re-consideration of resection after multidisciplinary discussion, in some selected cases, may confer some benefit [77,78,79].

## 7. Germline Testing and Tumor Gene Profiling

It is not currently recommended to perform tumor multigene NGS in patients with advanced PDAC in daily clinical practice [77,78,79,80]. According to ESCAT, there are three level I genomic alterations in metastatic PDAC: (1) germline mutations of BRCA 1 and BRCA 2 with a prevalence of 1–4%; (2) MSI-high with a prevalence of 1–3%; and (3) NTRAK fusions with a prevalence <1%. Olaparib maintenance therapy can be considered for metastatic PDAC patients with germline BRCA1 or BRCA2 mutations who have not progressed for more than 4 months after first-line platinum-based therapy [54]. For metastatic PDAC patients with MSI-high characteristics, the FDA has approved the anti-PD-1 immune checkpoint inhibitor pembrolizumab [81]. Further, tyrosine kinase inhibitors, such as larotrectinib and afatinib, might be considered in cases of tumor NTRK and NRG1 (up to 6%) gene fusions [82,83]. Up to 90–95% of PDAC has an activating point mutation in the KRAS oncogene [84,85]. Some authors consider the determination of KRAS status important, as patients with wild-type KRAS pancreatic tumors represent a distinct subgroup that may benefit from further molecular profiling with a higher probability of discovering targetable mutations [84,85].

## 8. Special Conditions

### 8.1. Germline BRCA Mutations

Patients with defects in DNA damage response (DDR) genes causing homologous recombination deficiency (HRD) represent a clinically relevant subgroup of PDAC patients with potential therapeutic implications. The better-characterized HRD genes are BRCA1, BRCA2, PALB2, ATM, RAD51, CHEK2, ATR and FANC genes [86]. Accumulating evidence from non-randomized clinical trials has identified HRD as an important biomarker of possible therapeutic response for platinum-based chemotherapy; therefore, FOLFIRINOX is the preferred first-line regimen for PDAC patients with this condition [86,87]. Moreover, maintenance with Olaparib might be offered if available due to regulatory restrictions [54].

### 8.2. Pancreatic Acinar Cell Carcinoma

Pancreatic acinar cell carcinoma (PACC) represents just 0.2–2% of all pancreatic malignancies in adults and is generally characterized as having a better prognosis [88,89]. Randomized trials are missing, though it seems that FOLFIRINOX might be the preferred first-line regimen [90,91,92,93]. Unlike in PDAC, metastasectomy is sometimes performed in conjunction with resection of the primary tumor. Molecular testing for all PACC patients for somatic mutations should be considered as these occur at higher incidences and are frequently actionable. Further, a higher rate of MSI-H/dMMR has been described in PACC [88].

### 8.3. Adenosquamous Carcinoma of the Pancreas

Adenosquamous carcinoma of the pancreas (ASCP) comprises 0.38–10% of exocrine pancreatic cancers [88,94,95] and is considered more aggressive than PDAC. According to the limited retrospective studies, there is no preferred regimen in a first-line setting, and both FOLFIRINOX and gemcitabine/nab-paclitaxel have shown efficacy [88,96,97]. In particular, according to a multicenter retrospective analysis of 116 patients with metastatic or recurrent ASCP treated with first-line chemotherapy, median OS, median PFS and objective response rates were 7.3 months, 2.8 months and 26.9% in patients treated with gemcitabine/nab-paclitaxel and 7.2 months, 2.3 months and 20.0% in patients treated with FOLFIRINOX [84].

## 9. Future Directions and Conclusions

Substantial progress in recent years has dramatically increased our knowledge of the molecular basis of many different types of solid tumors, revealing new potential therapeutic targets for more effective personalized anticancer treatments. Unfortunately, PDAC lags behind this success, with only a very modest benefit in survival outcomes. A severe lack of early diagnosis coupled with resistance to the most available therapeutic options renders pancreatic cancer a major clinical concern. Novel emerging diagnostic and predictive/prognostic biomarkers are needed. miRNA profiling seems to be a promising biomarker but has not been used in clinical trials to date [98,99,100,101]. Although it has traditionally been approached as one disease, accumulated evidence points to the clinical heterogeneity of this disease, which translates into disparity in outcomes between patients. Nowadays, the ability to identify transcriptional molecular subtypes of pancreatic adenocarcinomas has become a reality, but this binary classification (classical and basal-like subtypes) of tumor cells has failed to faithfully recapitulate the complexity of PDAC (heterogeneity and plasticity) and is not used in clinical practice [9]. The vast majority of PDAC patients harbor KRAS somatic mutations. KRAS codon 12 mutations are more common and constitute about 71% of all cases. These alterations include G12D (42%), G12V (32%), G12R (15%), G12C (1.5%), G12A (0.4%) and G12S (0.1%) [102]. Recently, KRAS-targeted therapeutics have been developed, and some early phase trials have shown encouraging results in metastatic settings (Sotorasib, Adagrasib) [103,104,105]. Historically, PDAC (excluding MSI-high tumors) did not show any response to immunotherapy and is, therefore, considered an immunologically “cold” tumor. However, there are some studies that focus on making tumors immunologically active by activating innate lymphocytes or creating an inflammatory response in the TME, thereby activating cytotoxic T cells. Immunotherapy is under investigation with different chemotherapy regimens, PARP inhibitors and target therapies [2,6,102]. Regarding chimeric antigen receptor T-cell (CAR-T) therapy in PDAC, the main limitation corresponds to antigen selection since they can have variable or heterogeneous expression in cancer cells, determining an elevated risk of toxicity [106,107,108]. Another interesting target in PDAC is the Claudin family. Different types of Claudin proteins play an important role in the EMT progression of pancreatic malignant and benign tumors, tumor development, nerve infiltration, tissue infiltration and metastatic implantation, and we await the results from clinical trials enrolling Claudin-18.2-positive PDAC patients [109]. Interestingly, recent studies have described that microbiota also contributes to cancer onset and progression by activating oncogenic signaling, enhancing oncogenic metabolic pathways, altering cancer cell proliferation and triggering chronic inflammation that suppresses tumor immunity [110]. However, ongoing research into the understanding of the complex interplay between the tumor, stroma and tumor microenvironment is needed to better select agents targeting these compartments.
